# Sub-micron scale patterning of fluorescent silver nanoclusters using low-power laser

**DOI:** 10.1038/srep23998

**Published:** 2016-04-05

**Authors:** Puskal Kunwar, Jukka Hassinen, Godofredo Bautista, Robin H. A. Ras, Juha Toivonen

**Affiliations:** 1 Tampere University of Technology, Department of Physics, Tampere, FI-33101, Finland; 2 Aalto University, Department of Applied Physics, Espoo, FI-02150, Finland

## Abstract

Noble metal nanoclusters are ultrasmall nanomaterials with tunable properties and huge application potential; however, retaining their enhanced functionality is difficult as they readily lose their properties without stabilization. Here, we demonstrate a facile synthesis of highly photostable silver nanoclusters in a polymer thin film using visible light photoreduction. Furthermore, the different stages of the nanocluster formation are investigated in detail using absorption and fluorescence spectroscopy, fluorescence microscopy, and atomic force microscopy. A cost-effective fabrication of photostable micron-sized fluorescent silver nanocluster barcode is demonstrated in silver-impregnated polymer films using a low-power continuous-wave laser diode. It is shown that a laser power of as low as 0.75 mW is enough to write fluorescent structures, corresponding to the specifications of a commercially available laser pointer. The as-formed nanocluster-containing microstructures can be useful in direct labeling applications such as authenticity marking and fluorescent labeling.

Metal nanoclusters are ultrasmall particles with diameters of typically less than 2 nm. Such nanoclusters have significantly different optical, electrical and chemical properties compared to their larger counterparts and act as a bridge between isolated metal atoms and nanoparticles^1^,^2^. Furthermore, these nanoclusters possess molecular properties like strong absorption and fluorescence due to discretization of energy levels as the size of the nanoclusters decreases down to few atoms^1^,^2^,^3^,^4^. The fluorescence is observed as a broadband emission with large Stokes shift and high fluorescence quantum yield^5^,^6^. Thus, metal nanoclusters have been proposed for many applications such as sensing, imaging, data storage and labeling^3^,^4^.

Silver nanoclusters are often synthesized and studied in the solvated state. These nanoclusters can be prepared by reducing silver salts by chemical^6^,^7^,^8^, electrochemical^9^, radiolytic^10^, sonochemical^11^, or photoreduction^4^,^12^ methods in a scaffold that stabilizes the growth of nanoclusters and prevents formation of larger nanoparticles. For example, synthetic polymers, DNA, dendrimer and some small molecules containing thiols and carboxylic groups can act as the stabilizing scaffold^1^,^2^,^3^,^4^. There is an abundance of literature about light-induced formation of silver nanoclusters using traditional photographic processes, where light reduces the silver ions into unstabilized silver clusters^13^,^14^ and larger particles that are non-fluorescent. Similar to these processes, stabilized silver nanoclusters have been formed by direct laser writing (DLW) to create various fluorescent 2D and 3D structures^15^,^16^,^17^,^18^. Multiphoton DLW has been shown to produce photostable silver nanoclusters in glass, zeolites^15^,^16^,^17^, and, more recently, in poly(methacrylic acid) (PMAA) thin films^18^.

Despite the proven potential of multiphoton DLW, this method requires an expensive excitation source, often a femtosecond laser, which delivers a very high laser intensity^19^,^20^,^21^. Obviously, the expenses and complexity of the system can be greatly reduced by using single-photon DLW, which utilizes low-cost light sources such as continuous wave (CW) lasers for fabrication. Furthermore, this reduces significantly the light intensities required to pattern structures^22^ as compared to multiphoton DLW. DLW can be utilized to fabricate fluorescent micro-labels that are important elements in imaging, cell labeling, authenticity marking and fluorescent tagging^23^. However, the micro-labels based on organic fluorescent dyes usually degrade quickly due to inherent photobleaching and reduced photostability^24^,^25^. Hence, the development of fabrication methods for the production of highly photostable micro-labels is timely and very important.

In this Article, a straightforward synthesis and stabilization of Ag nanoclusters in a polymer thin film is demonstrated using direct absorption-based laser exposure. The different stages of formation and photobleaching of the nanoclusters are studied in detail using absorption and fluorescence spectroscopy, fluorescence microscopy, and atomic force microscopy (AFM). In addition, DLW is utilized to fabricate fluorescent micron-sized quick response (QR) codes that can find applications, e.g., in authenticity marking applications. The as-formed Ag nanocluster micro-labels have a unique emission signature and good photostability under strong illumination. They were also found to be stable in ambient lighting conditions over the testing period of five weeks.

## Results

### Optimization of Ag/MAA ratio

In this study, we used Ag@PMAA thin films with a 50% Ag/MAA ratio. Previously, we have verified that samples containing Ag/MAA ratios that are greater than 75% are not optimal due to unwanted crystallization in the film^18^. Furthermore, the fluorescence intensity obtained from the written structure in the Ag@PMAA film gradually increases when the Ag/MAA ratio increases from 10% to 75% (see Supplementary Fig. S1). Even though the fluorescence yield in the structures written on the 75% Ag@PMAA samples is better than the structures written on 50% Ag@PMAA samples, the Ag/MAA ratio of 50% was considered optimized as it was below the crystallization limit.

### Formation and photobleaching of silver nanoclusters

**Figure 1:**
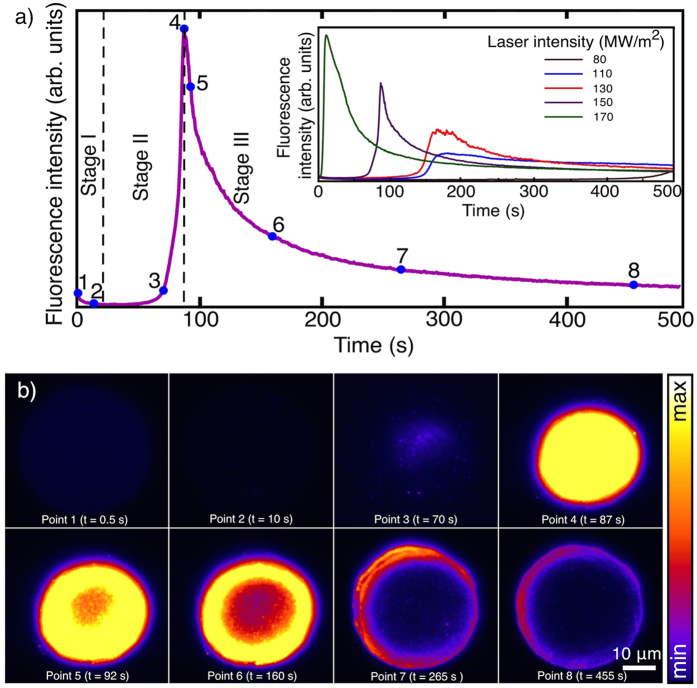
(**a**) Fluorescence intensity of the Ag@PMAA film during continuous laser irradiation with laser intensity of 150 MW m^−2^ and wavelength of 532 nm. Inset shows the fluorescence behavior using five different laser intensities. (**b**) Series of fluorescence microscopy images obtained at exposure points 1–8, which are marked by blue points in Fig. 1a).

The formation of nanoclusters was monitored by continuously recording the fluorescence spectra when a Ag@PMAA film with 50% Ag/MAA ratio was irradiated with a laser beam intensity of 150 MW m^−2^ and wavelength of 532 nm. The total emission intensities were estimated by integrating the areas under the curves of the fluorescence emission spectra between the wavelengths 600 nm to 700 nm. As observed in Fig. 1a, the formation and photobleaching of nanoclusters can be described qualitatively in three stages. The first stage is marked by a drop in the fluorescence intensity at the moment of exposure that we attribute to the photobleaching of unstable nanoclusters. These nanoclusters are likely formed in the solution prior to thin film preparation. As shown previously, such nanoclusters can be kinetically trapped in the film during spin coating and thus are not properly protected by MAA units^18^. The second stage is marked by a sharp increase in fluorescence intensity due to the formation of highly photostable silver nanoclusters. In the third stage, the fluorescence decreases exponentially as a result of photobleaching of nanoclusters. The bleaching rate is fast here due to the same laser intensity used for both exposure of the nanoclusters and excitation of the fluorescence. For reference, we performed a similar experiment at different input laser intensities and monitored the behavior of the detected fluorescence [inset of Fig. 1a]. As expected, the rates of formation and photobleaching of Ag nanoclusters are faster when higher laser intensities are used.

Figure 1b shows a series of fluorescence microscopy images obtained at different exposure points as marked in Fig. 1a. These exposure points define particular states of the formation and bleaching of the nanoclusters. At exposure point 1, the unstable nanoclusters emit weak fluorescence that bleaches to almost zero by exposure point 2. The formation of the new fluorescent nanoclusters starts at exposure point 3. From this point, the fluorescence intensity reaches its maximum at exposure point 4. The exposure points from 5 to 8 indicate different stages of the photobleaching from the beginning of the bleaching until to almost complete loss of the fluorescence. For laser intensity of 150 MW m^−2^ and wavelength of 532 nm, the exposure points from 1 to 8 correspond to 0.5 s, 10 s, 70 s, 87 s, 92 s, 160 s, 265 s and 455 s from the beginning of the irradiation, respectively. Hence, similar to Fig. 1a, the different stages of formation and photobleaching of the nanoclusters are qualitatively shown in Fig. 1b. This figure also shows that the bleaching occurs radially from the center towards the edges and mainly follows the Gaussian intensity profile of the irradiating laser beam. A complete time series of the fluorescence behavior under continuous laser irradiation can be found as a video in the Supplementary information.

**Figure 2:**
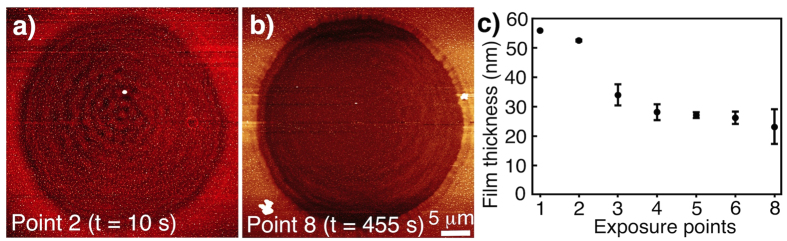
AFM images of the exposed area corresponding to exposure points (**a**) 2 and (**b**) 8. (**c**) Measured film thicknesses at different exposure points.

AFM was performed to examine the topographical changes in the areas exposed to the laser beam stopped at specific exposure points. Essentially, the AFM images at different stages of nanocluster formation have similar features. AFM images corresponding to the exposure points 2 and 8 are shown in Fig. 2. There is a circular indentation in the exposed area with interference fringes of the laser visible in all of the AFM images. Figure 2c shows the change in the film thickness of the exposed part at different exposure points. The thickness of the film before laser exposure was estimated to be 56 nm. This thickness decreases to almost half of the initial thickness when irradiated to exposure point 4. It is worth noting from Fig. 1 that the fluorescent intensity reaches its maximum at this exposure point. If the irradiation is continued, there is no further decrease in film thickness signifying that the conformational change of the polymer and/or ablation occurs during the formation of the nanoclusters and subsequently the film remains stable.

**Figure 3:**
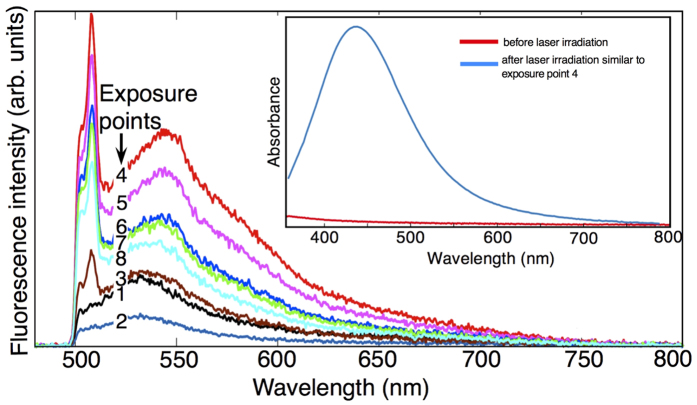
Emission spectra recorded at different exposure points of the nanocluster formation during the irradiation of Ag@PMAA film with laser beam of wavelength 473 nm and intensity of 70 MW m^−2^. Exposure points from 1 to 8 correspond to 0.5 s, 7 s, 21 s, 42 s, 50 s, 140 s, 270 s and 475 s from the beginning of the irradiation, respectively. The inset figure shows the absorption spectrum of Ag@PMAA film before (red curve) and after laser irradiation similar to exposure point 4 (blue curve).

Figure 3 shows the emission spectra at different stages of nanoclusters formation, which were recorded by exciting the sample using a 473 nm laser with an intensity of 70 MW m^−2^. The emission spectrum at the beginning of the laser exposure (at exposure point 1) is characterized by a broadband emission ranging from 500 nm to 800 nm, with an intensity maximum at 530 nm. Wavelengths shorter than 500 nm cannot be detected due to setup limitations (e.g. cutoff wavelengths of optical filters). As the irradiation is continued to exposure point 2, the intensity of the background fluorescence is decreased but there is no change in the shape of the spectrum. At exposure point 3, the emission slightly redshifts and a sharp peak at 510 nm starts to appear. The peak has been identified earlier as an enhanced Raman scattering^18^. At exposure point 4, the emission intensity reaches its maximum with peak wavelength at 545 nm and a broad shoulder is observed at 580 nm. The redshift in emission wavelength is attributed to changes in exposure conditions that modify the material properties. Our previous work with two-photon laser writing resulted a fluorescence maximum at 560 nm using the same material system^18^. While further studies are needed to clarify the microscopic origin of the wavelength shift, it is highly possible that the shift in fluorescence maximum is related to the exposure conditions. From exposure point 5 onwards, the emission maximum blueshifts and the shoulder at 580 nm becomes less prominent, finally disappearing at exposure point 8. As expected, there is no change in the position of the enhanced Raman peak at the different exposure points. Close inspection of the fluorescence spectra reveals that the fluorescence intensity, spectral line-width and peak wavelength change at different stages in a certain trend. From exposure point 1, they decrease by a small amount to exposure point 2, then increase to maximum at exposure point 4 and finally decrease until exposure point 8 (see Supplementary Fig. S2). Figure 3 inset shows an absorption spectrum obtained from a large area (1 mm × 1 mm) exposed to laser irradiation similar to exposure point 4, i.e., after the formation of photostable nanoclusters. The broad absorption spectrum has a maximum at 435 nm and shows similarities with the absorption spectra of Ag@PMAA nanoclusters in solution as reported previously^11^,^12^,^26^. On the other hand, the unexposed Ag@PMAA film does not show any noticeable absorption features at the 400–500 nm wavelength range.

### Fabrication of photostable micro-label

Prior to patterning micro-labels, we studied the dependence of the line-width and fluorescence intensity obtained from the structures written with different laser writing intensities. We found that both the line-width and fluorescence intensity increase with increasing laser intensities and the results are described in detail in the Supplementary information (see Supplementary Figs. S3, S4 and S5).

**Figure 4:**
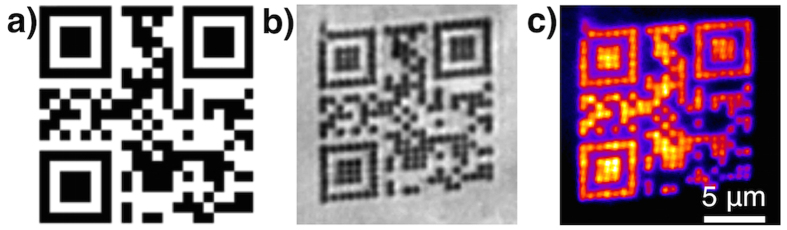
(**a**) Target image for patterning a QR code. (**b**) Bright field microscopy image of the fabricated QR-code in the Ag@PMAA sample. (**c**) Corresponding fluorescence microscopy image of the same area. Fluorescence microscopy images were recorded using a LED light source with an excitation wavelength of 470 nm and an excitation intensity of 2 MW m^−2^.

A DLW setup, described in the *Methods* section, was used to fabricate a photostable micro-label shown in Fig. 4. In order to write the pattern shown in Fig. 4a, the sample was scanned against a fixed focused laser beam that is controlled with a shutter to expose black pixels of the pattern and turn off the laser power for the white pixels. The written structure can be read with a fluorescence microscope immediately after the laser writing without any post-processing. The fabricated structure shown in Fig. 4b,c was written with a laser writing intensity of 45 GW m^−2^ (laser power of 0.75 mW at the back aperture of the objective), a laser writing wavelength of 405 nm and an exposure time of 0.22 s at each irradiated pixel in 50% Ag@PMAA samples. It took about 100 s to write the complete QR code; however, the exposure time and the laser intensity can be further adjusted to decrease the time needed to fabricate the code and to improve the resolution of writing. The resolution of this fabrication technique is diffraction limited (see Supplementary Figs. S3 and S4) and is comparable to single-photon-absorbed polymerization. The fluorescence image shown in Fig. 4c was taken with a custom-built fluorescence microscope equipped with a LED light source with an excitation wavelength of 470 nm and an excitation intensity of 2 MW m^−2^. The fluorescence obtained from the written micro-label is attributed to the formation of Ag nanoclusters. The broadband emission and sharp peak of Raman scattering at around 510 nm makes the spectral signature unique. Furthermore, the good correspondence of the fabricated micro-label and the target suggests the feasibility of this method and has great potential for applications like authenticity marking and fluorescence tagging.

### Stability of silver nanoclusters

The photostability of the as-formed fluorescent micro-label was investigated by recording the emission spectra from an area containing the micro-label, an area unexposed to writing beam and a PMAA film containing an organic dye Rhodamine 6G. The samples were irradiated with a laser beam of wavelength of 473 nm and laser intensity of 2 MW m^−2^ for 180 s. The fluorescence intensity was measured by integrating the area under the emission curve and plotted as a function of time (see Supplementary Fig. S6). The unexposed area shows a rapid photobleaching resulting in 7% of its initial emission intensity at the end of 3 minutes. However, the emission intensity from the area containing the micro-label decreased only to 40% of initial intensity under similar conditions. The as-formed Ag nanoclusters were also found to be highly photostable compared to that of a conventional fluorescent dye, Rhodamine 6G. The Rhodamine 6G sample bleaches even faster than area unexposed to the writing beam, resulting in a 3% of its initial emission intensity at the end of 180 seconds.

Finally, the stability of the fabricated micro-labels was investigated over time to test if the written structures and background are undergoing any chemical changes in time. Here, the micro-labels were stored in the dark at ambient room conditions for a period of six weeks. The fluorescence spectra were recorded weekly by exciting the micro-labels with a laser wavelength of 473 nm and laser intensity of 2 MW m^−2^. The fluorescence intensity was found to remain stable during the period of six weeks. Furthermore, we also found that the written structures stored under ambient room lighting for five weeks were still brightly fluorescent. These results imply that the fabricated micro-labels are photostable and also chemically stable over time.

## Discussion

Photostable silver nanoclusters have been synthesized and stabilized in PMAA films using a low power laser. As evidenced by absorption spectroscopy, fluorescence microscopy and spectroscopy, and AFM results, the formation of the nanoclusters can be divided into three stages. The first stage is the photobleaching of the unstable nanoclusters, which are likely formed during the sample preparation. We consider the formation of unstable nanoclusters based on the fact that the shape of the emission spectrum (Fig. 3, exposure point 1) obtained from these unstable nanoclusters is similar to emission spectrum of silver nanoclusters formed after laser activation; however, the spectral signature does not contain the Raman scattering. Nevertheless, the existence of similar unstable nanoclusters was also reported before^18^,^27^. The second stage is the formation of photostable brightly fluorescent nanoclusters. The third stage is marked by exponential decay of fluorescence due to photobleaching of nanoclusters. The applicability of this technique is demonstrated by fabricating fluorescent micro-labels in PMAA film by using DLW. The as-formed micro-labels are highly photostable compared to an organic dye, Rhodamine 6G. These nanoclusters do not lose fluorescence when stored at dark ambient conditions at least for the investigated period of six weeks. Altogether, the cost-effective fabrication of silver nanocluster-based micro-labels presented here essentially provides a microscopic two-level authentication platform, which are unique, and photostable emission signatures and 2D arbitrary patterning. As this technique uses very low power laser, it is anticipated that this technique can be further enhanced to a cost-effective parallel fabrication by performing, e.g., holographic or shaped light exposure.

## Methods

### Materials and sample preparation

The polymer used in this experiment is poly(methacrylic acid) (PMAA) (PolySciences, M_w_ = 100, 000), which is a well-known stabilizing agent for brightly fluorescent silver nanoclusters in solution. The source of the silver ions is silver nitrate (AgNO_3_) (Sigma-Aldrich, >99,8%), which readily forms silver nanoclusters in the aqueous solution of PMAA upon visible light exposure. These two chemicals were mixed to obtain PMAA concentration of 1.5 w-% and Ag/MAA ratios ranging from 0% to 75%. The borosilicate coverslips (22 mm × 22 mm × 0.17 mm) were used as substrates for the thin films. The solutions were spin-coated on glass substrates at 1500 RPM for 120 s. The samples were dried in vacuum and care was taken to prevent their exposure to ambient light. The Rhodamine 6G sample was prepared by spin coating the solution, which is made by mixing 10 nM Rhodamine 6G in 1.5 w-% PMAA polymer.

### Laser writing setup

The experimental setup used in DLW of fluorescent nanocluster microstructures is shown in Supplementary Fig. S7. This setup consists of continuous-wave (CW) laser sources, achromatic doublet lenses (L1, L2, L3, L4 and L5), dichroic mirrors (DM), an objective lens (HCX PL APO, 1.4 NA, 100×, Leica), translation stage for sample scanning (Nanomax, Thorlabs), and a camera (DCC1545M, Thorlabs). The light source used was 405 nm CW diode laser (DL 100, TOPTICA Photonics), 473 nm diode laser (RLTMBL-473-150-10, Roithner Lasertechnik GmbH) or 532 nm CW semiconductor laser (Millennia Pro, Spectra Physics) depending on the application. The assembly of 40 mm and 200 mm achromatic lenses (L1 and L2) collimated and expanded the laser beam. The shutter (SH05, Thorlabs) was placed after the beam expander. This beam was directed towards the shortpass dichroic mirror. The dichroic mirror (BLP01-488R, Semrock) was used to guide 473 nm and 405 nm laser beams; however, this dichroic mirror was replaced by another dichroic mirror (FF535-SDi01, Semrock) while using 532 nm laser beam. These dichroic mirrors allow the passage of the laser beam towards the objective lens. The objective lens focused the laser beam into the sample. A brightfield microscopy setup was added to image the laser writing process and obtain a bright field image. Lens L3 can be added in between M2 and DM to focus the light on back aperture of objective, which, in turns, defocuses the laser beam, thereby exposing a large circular area in the sample.

### Fluorescence microscopy and spectroscopy

The fabricated structures were imaged and characterized using a custom-built fluorescence microscope, which is shown in Supplementary Fig. S8. The setup contains a 473 nm diode laser (RLTMBL-473-150-10, Roithner Lasertechnik GmbH), a 470 nm LED (Osram), an excitation filter (FF02-470/100, Semrock), two achromatic lenses (f = 200 mm, Thorlabs), a dichroic mirror (FF484-Fdi01, Semrock), objective lens (HCX PL APO, 1.4 NA, 100×, Leica), a manually controlled stage (Nanomax, Thorlabs), an emission filter (BLP01-488R-25, Semrock), a flip mirror, a spectrometer (Avaspec 2048, Avantes) and an EMCCD camera (iXon3 897, Andor). The excitation light was guided towards the objective lens with the help of shortpass dichroic mirror (BLP01-488R, Semrock). The convex lens L1 focused the laser beam into the back aperture of the objective lens, thus illuminating a larger area of the sample. The defocused beam excites the written structure, which, in turn, produces the fluorescence signal. This signal is collected by an objective lens and imaged with a camera. An Avantes spectrometer was added to measure the emission spectrum from the sample. The fluorescence images were false-colored based on their raw values using ImageJ software.

### Absorption spectroscopy

The absorption spectrum of the fabricated structures was measured with UV-VIS-NIR spectrophotometer (PerkinElmer Lambda 950). The spectra from the unexposed part was also measured and subtracted from the spectra of written structures to obtain the final spectra of the silver clusters in the written structures.

### AFM imaging

Veeco Dimension 5000 AFM with Nanoscope V controller was employed in tapping mode for the detailed analysis of written structures. The thickness of the film was estimated by scratching the sample with the scalpel and by scanning the AFM tip perpendicular to the scratch. AFM was also used to measure the detailed topographical features of the area exposed to the writing beam.

## Additional Information

**How to cite this article**: Kunwar, P. *et al*. Sub-micron scale patterning of fluorescent silver nanoclusters using low-power laser. *Sci. Rep.* **6**, 23998; doi: 10.1038/srep23998 (2016).

## Supplementary Material

Supplementary Information

Supplementary video
