# Supplementary material for: Sub-micron scale patterning of fluorescent silver nanoclusters using low-power laser
Source: Sci Rep. 2016 Apr 5;6:23998. doi: 10.1038/srep23998 (PMC4820741; doi:10.1038/srep23998)
Supplement: Supplementary Information [file srep23998-s1.pdf]

Sub-micron scale patterning of fluorescent silver nanoclusters using low-power laser

Puskal Kunwar[1,*], Jukka Hassinen[2], Godofredo Bautista[1], Robin H. A. Ras[2], and Juha Toivonen[1]

[1]Tampere University of Technology, Department of Physics, Tampere, FI-33101, Finland

[2]Aalto University, Department of Applied Physics, Espoo, FI-02150, Finland

*puskal.kunwar@tut.fi

Table of Contents

1. Effect of silver concentration on fluorescence intensity of written structures
2. Dependence of intensity, spectral line-width and peak wavelength of fluorescence spectra at different stages of nanocluster formation
3. Dependence of line-width and fluorescence intensity of microstructures written using different laser writing intensities
4. Photostability of written structures
5. Experimental setups
6. Supplementary video
7. Supplementary references

Effect of silver concentration on fluorescence intensity of written structures

Concentration test was performed to investigate the effect of silver content on the origin of fluorescence from silver nanocluster microstructures. The concentration test was done by recording the emission spectra from structures written on samples with different Ag/MAA ratios varying from 10% to 75 %. Ag/MAA ratios greater than 75% were not considered for study due to unwanted crystallization in the film. The structures were written with laser writing intensity of 45 GW m^{-2}, wavelength of 405 nm and scanning speed of 10 μm s^{-1}. Emission spectra were obtained by exciting the structures with a laser beam of wavelength 473 nm and intensity of 2 MW m^{-2} (Figure S1). As seen in Figure S1, the fluorescence signal is highest for 75% Ag@PMAA sample and almost zero for 10% Ag@MAA samples. We were unable to write structure in the sample with no silver content. This experiment ensures that the fluorescence is primarily related to the presence of silver in the polymer, which is consistent with our previous results in two-photon writing of nanoclusters[S1].

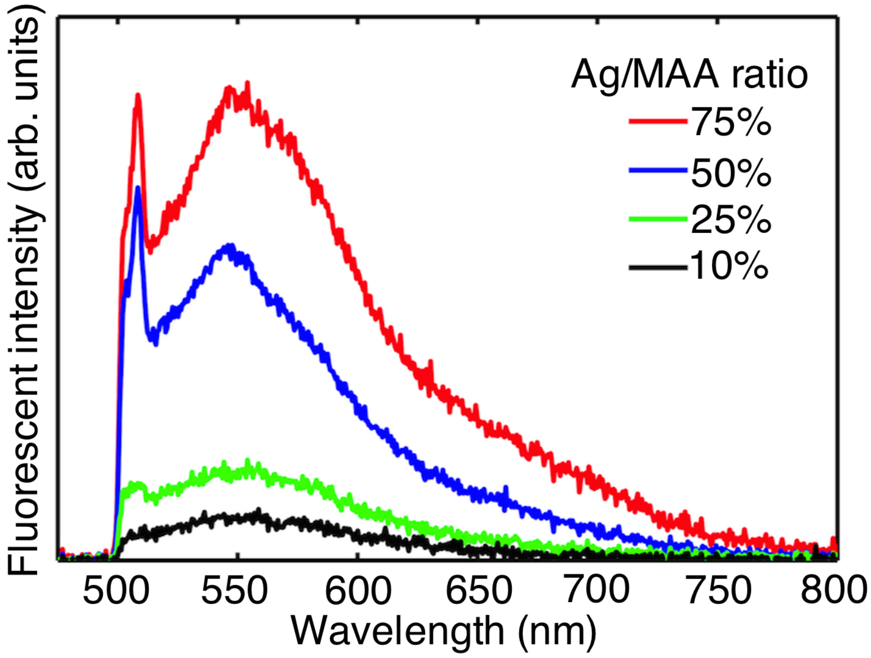

Figure S1. Fluorescence spectra obtained from the laser-written silver nanoclusters in Ag@PMAA film with different Ag/MAA concentration ranging from 10% to 75% when excited with excitation wavelength of 473 nm laser and laser intensity of 2 MW m^{-2}.

Compared to Figure S1, it is worth noting that there is blue-shift in emission spectra, which are shown in Figure 3. The blue-shift can originate from the significant differences in the laser exposure and is also observed compared to our previous work[S1]. Note the laser writing intensities, laser writing wavelength and exposure time are different for these experiments. As shown in Figure S2, such changes in the exposure parameters cause variation in the emission properties of nanoclusters. However, further experiments are still needed to verify the molecular level origins of such variations in the properties of nanoclusters, which can originate from differences in the nanocluster size and geometry, oxidation state, or environment/stabilizing matter [S2,S3].

Dependence of intensity, spectral line-width and peak wavelength of fluorescence spectra at different stages of nanocluster formation

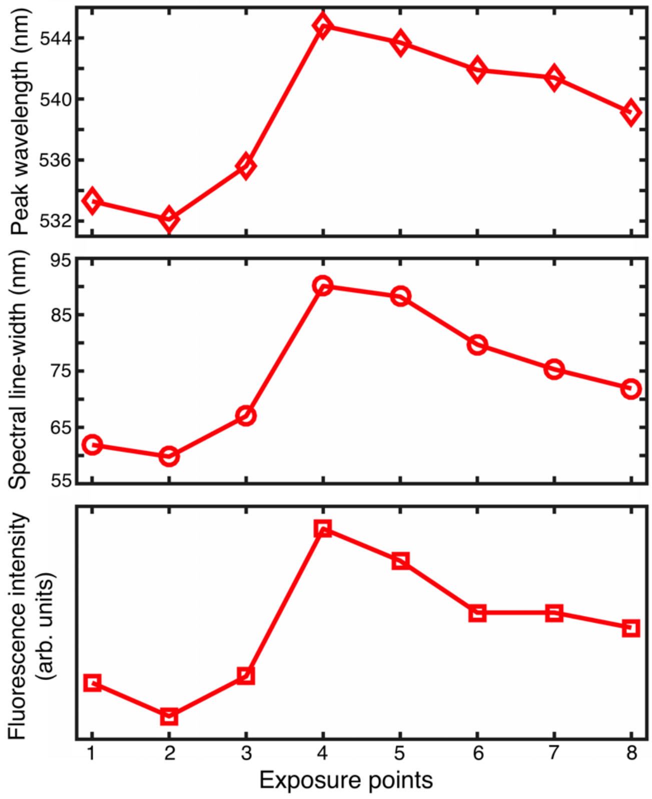

Figure S2. Line graphs showing the change in fluorescence intensity, spectral line-width and peak wavelength at different stages of Ag@PMAA nanocluster formation.

Dependence of line-width and fluorescence intensity of microstructures written using different laser writing intensities

In order to investigate the dependence of line-width and fluorescence intensity on laser writing intensity, similar arrays of lines were written with different laser writing intensities ranging from 7.5 GW m^{-2} to 90 GW m^{-2}, laser writing wavelength of 405 nm and scanning speed of 10 μm s^{-1}. The AFM images shown in Figure S3 were taken from structures written with 5 different laser-writing intensities of 7.5 GW m^{-2}, 15 GW m^{-2}, 30 GW m^{-2}, 60 GW m^{-2}, and 90 GW m^{-2}. The line-widths of the written structures were estimated from the line profiles measured across the written structures and are shown in Figure S4. The estimated line-widths are 255 nm, 285 nm, 415 nm, 805 nm, and 930 nm, respectively.

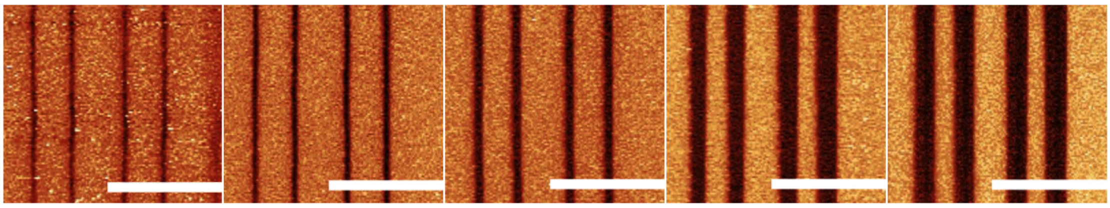

Figure S3. AFM images of structures written with different laser writing intensities of 7.5 GW m^{-2}, 15 GW m^{-2}, 30 GW m^{-2}, 60 GW m^{-2}, and 90 GW m^{-2} (from left to right), laser wavelength of 405 nm, and scanning speed of 10 μm s^{-1}. Scale bars = 5 μm.

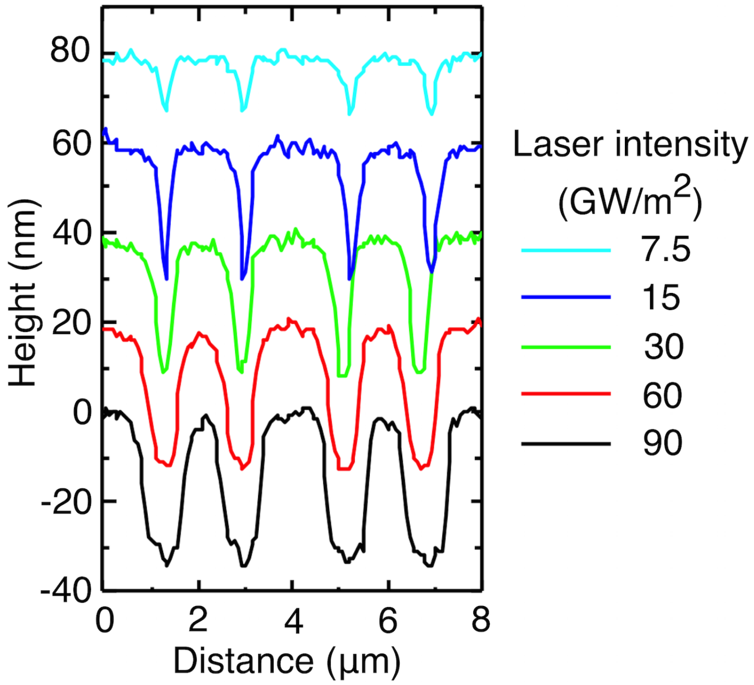

Figure S4. Height profiles (across the line) of structures written with different laser writing intensities of 7.5 GW m^{-2}, 15 GW m^{-2}, 30 GW m^{-2}, 60 GW m^{-2}, and 90 GW m^{-2}.

Fluorescence spectra were recorded from similar arrays of lines written with different laser writing intensities as shown in Figure S3. The spectra were recorded by exciting the structures with laser intensity of 2 MW m^{-2} and wavelength of 473 nm (Figure S5). It is evident that the fluorescent intensity is higher for structures written with higher laser intensity and vice versa.

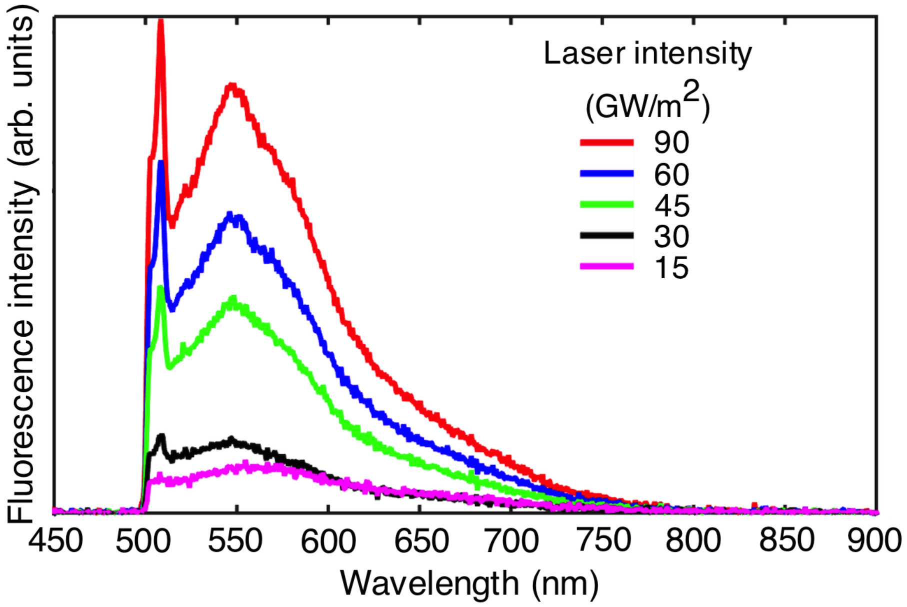

Figure S5. Fluorescence spectra recorded from the written structures with different writing laser intensities of 15 GW m^{-2}, 30 GW m^{-2}, 45 GW m^{-2}, 60 GW m^{-2}, and 90 GW m^{-2}. The fluorescence spectra were recorded by exciting the structures with laser intensity of 2 MW m^{-2} and wavelength of 473 nm.

Furthermore, the line profiles shown in Figure S4 show that the lowest laser intensity of 7.5 GW m^{-2} already ablates a part of the material. On the other hand, fluorescence spectra depicted in Figure S5 show that the structures, which are written with laser power above 15 GW m^{-2}, are fluorescent. This confirms that it is highly unlikely to create silver nanoclusters without conformational change and/or ablation process in this material system. This result is consistent with the results depicted in Figures 1b and 2.

Photostability of written structures

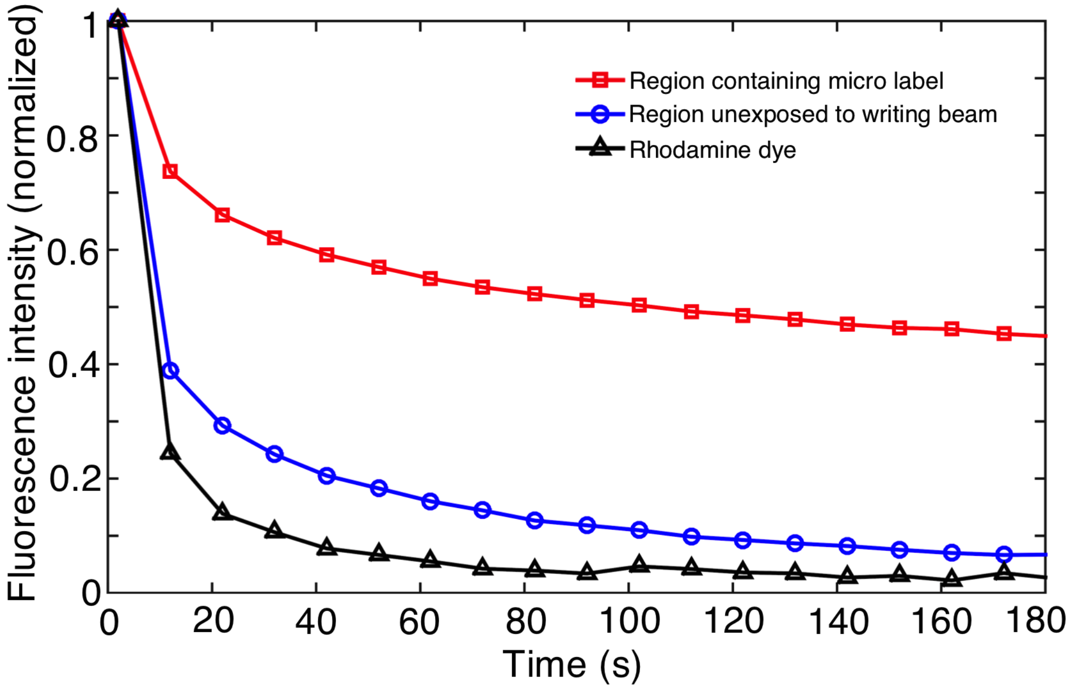

Figure S6. Normalized photobleaching curves of area containing written structures, area unexposed to writing beam and Rhodamine 6G dye containing PMAA film. The bleaching curves were recorded by exciting the structures with laser beam of wavelength 473 nm and laser intensity of 2 MW m^{-2}.

Experimental setups

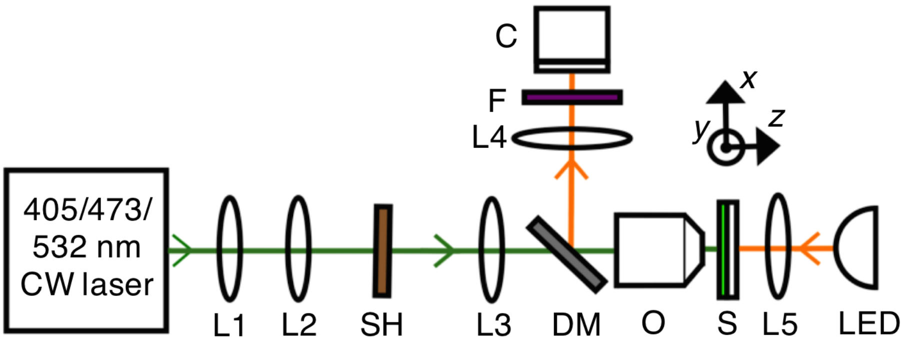

Figure S7. Schematic diagram of direct laser writing setup for patterning silver nanocluster microstructures. Components: lens (L), shutter (SH), dichroic mirror (DM), filters (F), camera (C), objective (O), sample mounted at translation stage (S) and red light emitting diode (LED) for bright field microscopy.

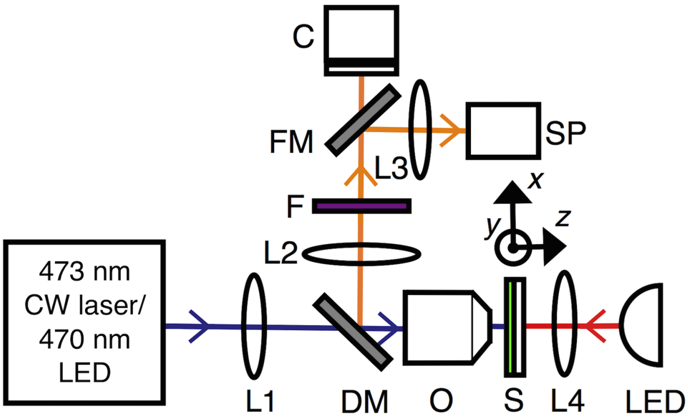

Figure S8. Schematic diagram of the fluorescence microscopy and spectroscopy setup. Components: lens (L), filter (F), dichroic mirror (DM), camera (C), flip mirror (FM), spectrometer (SP), objective (O), sample mounted at translation stage (S) and red light emitting diode (LED) for bright field microscopy.

Supplementary video. Formation and photobleaching of silver nanoclusters in poly(methacrylic acid) (PMAA) thin film. This video was recorded when a Ag@PMAA film with 50% Ag/MAA ratio was continuously irradiated with a laser beam intensity of 150 MW m^{-2} and wavelength of 532 nm. This movie shows a complete time series of the fluorescence behavior of silver nanoclusters under continuous laser irradiation.

Supplementary references

S1. Kunwar, P., Hassinen, J., Bautista, G., Ras, R. H. A., and Toivonen, J. Direct Laser Writing of Photostable Fluorescent Silver Nanoclusters in Polymer Films. *ACS Nano* **8**, 11165 (2014).

S2. Díez, I., Pusa, M., Kulmala, S., Jiang, H., Walther, A., Goldmann, A. S., Müller, A. H. E., Ikkala, O. & Ras, R. H. A. Color Tunability and Electrochemiluminescence of Silver Nanoclusters. *Angewandte. Chemie International Edition* **48**, 2122-2125 (2009).

S3. Díez, I., Ras, R. H. A., Kanyuk, M. I. & Demchenko, A. P. On Heterogeneity in Fluorescent Few-Atom Silver Nanoclusters. *Physical Chemistry Chemical Physics* **15**, 979-985 (2013).
